# The immunological mechanisms that control pneumococcal carriage

**DOI:** 10.1371/journal.ppat.1006665

**Published:** 2017-12-21

**Authors:** Simon P. Jochems, Jeffrey N. Weiser, Richard Malley, Daniela M. Ferreira

**Affiliations:** 1 Department of Clinicial Sciences, Liverpool School of Tropical Medicine, Liverpool, United Kingdom; 2 Department of Microbiology, New York University School of Medicine, New York, New York, United States of America; 3 Division of Infectious Diseases, Boston Children′s Hospital, Harvard Medical School, Boston, Massachusetts, United States of America; University of Basel, SWITZERLAND

## Abstract

Colonization of the human nasopharynx by pneumococcus is extremely common and is both the primary reservoir for transmission and a prerequisite for disease. Current vaccines targeting the polysaccharide capsule effectively prevent colonization, conferring herd protection within vaccinated communities. However, these vaccines cover only a subset of all circulating pneumococcal strains, and serotype replacement has been observed. Given the success of pneumococcal conjugate vaccine (PCV) in preventing colonization in unvaccinated adults within vaccinated communities, reducing nasopharyngeal colonization has become an outcome of interest for novel vaccines. Here, we discuss the immunological mechanisms that control nasopharyngeal colonization, with an emphasis on findings from human studies. Increased understanding of these immunological mechanisms is required to identify correlates of protection against colonization that will facilitate the early testing and design of novel vaccines.

## Introduction

*Streptococcus pneumoniae* (the pneumococcus) is the most common bacterial cause of pneumonia, meningitis, and otitis media in children [1]. Pneumococcal pneumonia is also associated with significant morbidity and mortality in the elderly [2]. Such increased pneumococcal disease rates in the elderly could be associated with an altered colonization niche, increased oropharyngeal carriage, as well as with alterations in immunity (reviewed by [3]). Moreover, pneumococcal pneumonia is a leading cause of death during seasonal and pandemic influenza infections [4]. In addition, pneumococcus is the most common cause of pneumonia, sepsis, and meningitis among those infected with HIV [5]. Pneumococcal disease is also increased with exposure to cigarette smoke and air pollution [6, 7]. A further concern of pneumococcal infection is high rates of resistance to multiple classes of frequently used antibiotics [8].

Stable colonization (i.e., carriage) within the human nasopharynx, the commensal state of *S*. *pneumoniae*, is extremely common because 40% to 95% of infants and 1% to 10% of adults are colonized at any time [9]. In children, simultaneous colonization with multiple pneumococcal strains is not uncommon [10]. Carriage is asymptomatic in adults but can be associated with mild rhinitis symptoms in children [11]. Importantly, carriage is the primary reservoir for transmitted pneumococci and is a prerequisite for pneumococcal disease [12]. Increased carriage density has been demonstrated to correlate with transmission within households [13]. Moreover, coinfection with influenza or HIV can lead to an increased density of colonizing pneumococci, which is associated with an elevated risk of pneumonia and mortality [14, 15]. An increased carriage density was confirmed as a risk factor for pneumococcal pneumonia in a second patient cohort of adult patients with radiologically confirmed community-acquired pneumonia [16]. While causality cannot be determined from these epidemiological studies, a high pneumococcal density in the nasopharynx may facilitate bacterial invasion and micro-aspiration into the lungs and thereby increase the likelihood of progression of infection to pneumonia [17].

In addition to being a prerequisite of disease and a reservoir for transmission, carriage also causes an increase in antibody levels against immunodominant pneumococcal surface antigens, including capsular polysaccharides and proteins, potentially immunizing against future colonization and infection [18, 19]. Moreover, carriage increases levels of pneumococcus-specific cluster of differentiation 4 (CD4^+^) T memory cells both in blood and in the lung [20]. Even among HIV-infected adults, recurrent disease caused by pneumococci of the same capsular polysaccharide serotype is extremely infrequent, which suggests a protective effect of exposure [21].

Three pneumococcal vaccine formulations are currently licensed, all of which induce humoral responses against a subset of the 98 currently described immunologically distinct capsular polysaccharide serotypes [22, 23]. The pneumococcal polysaccharide vaccine (PPV), which covers 23 serotypes, induces T cell–independent responses by B cells and plasma cells expressing anticapsular immunoglobulin G (IgG) in adults but is poorly immunogenic in young children [24]. The protective efficacy of this vaccine for nonbacteremic pneumonia remains controversial despite many years of use, and there are theoretical concerns about immunological harm resulting from B cell depletion [25]. The pneumococcal conjugate vaccine (PCV) couples capsular polysaccharides to a carrier protein, which elicits T-cell help and results in improved memory–B cell formation, affinity maturation, class switching, and levels of IgG. Two formulations that target 10 or 13 serotypes have been licensed to date [24]. Serotypes included in PCV10 are 1, 4, 5, 6B, 7F, 9V, 14, 18C, 19F, and 23F. The PCV13 formulation includes the additional serotypes 3, 6A, and 19A. An investigational vaccine, which also includes the two serotypes 22F and 33F in addition to those in PCV13, might increase this protection to 15 serotypes in the near future [26]. An important benefit of PCV-induced immunity is a decreased incidence of vaccine-type pneumococcal carriage in the population (herd protection). Since the release and widespread use of PCVs among children, a decrease in the circulation of the targeted serotypes in immunized populations has been observed. As a result, the incidence of invasive pneumococcal disease and pneumonia in both vaccinated children and unvaccinated adults has been reduced dramatically [27]. In fact, most of the overall efficacy of PCV has been attributed to herd immunity for populations at risk. Problems that remain with PCV are the high cost of this complex vaccine, evidence of gradual replacement by serotypes not covered by the vaccine, and poor matching to serotypes circulating in developing countries, which suffer the largest burden of disease [28]. Novel vaccination strategies are needed to complement current vaccination and treatment options. As a result, at least nine protein-based or whole-cell pneumococcal vaccines are currently in preclinical and clinical trials [29]. Nasopharyngeal carriage is an endpoint for several clinical trials testing novel vaccines because it is a fast, easy-to-measure, and cost-efficient surrogate for disease endpoints [30]. However, the immunological correlates of protection against carriage have not yet been identified in humans, which hinders the development of effective novel vaccines and does not provide a clear licensure pathway for protein-based vaccines.

Factors promoting pneumococcal colonization include, among others, evasion of mucociliary clearance, host nutrient availability, niche competition with other pneumococci and microbes of other species (reviewed in [31]), and the ability to avoid the host immune response long enough to allow successful transmission to a new host. Here, we focus on the immunological factors that control pneumococcal carriage, with an emphasis on mechanisms that have been described in animal models and subsequently examined in human studies.

## Capsule-specific antibodies

**Fig 1:**
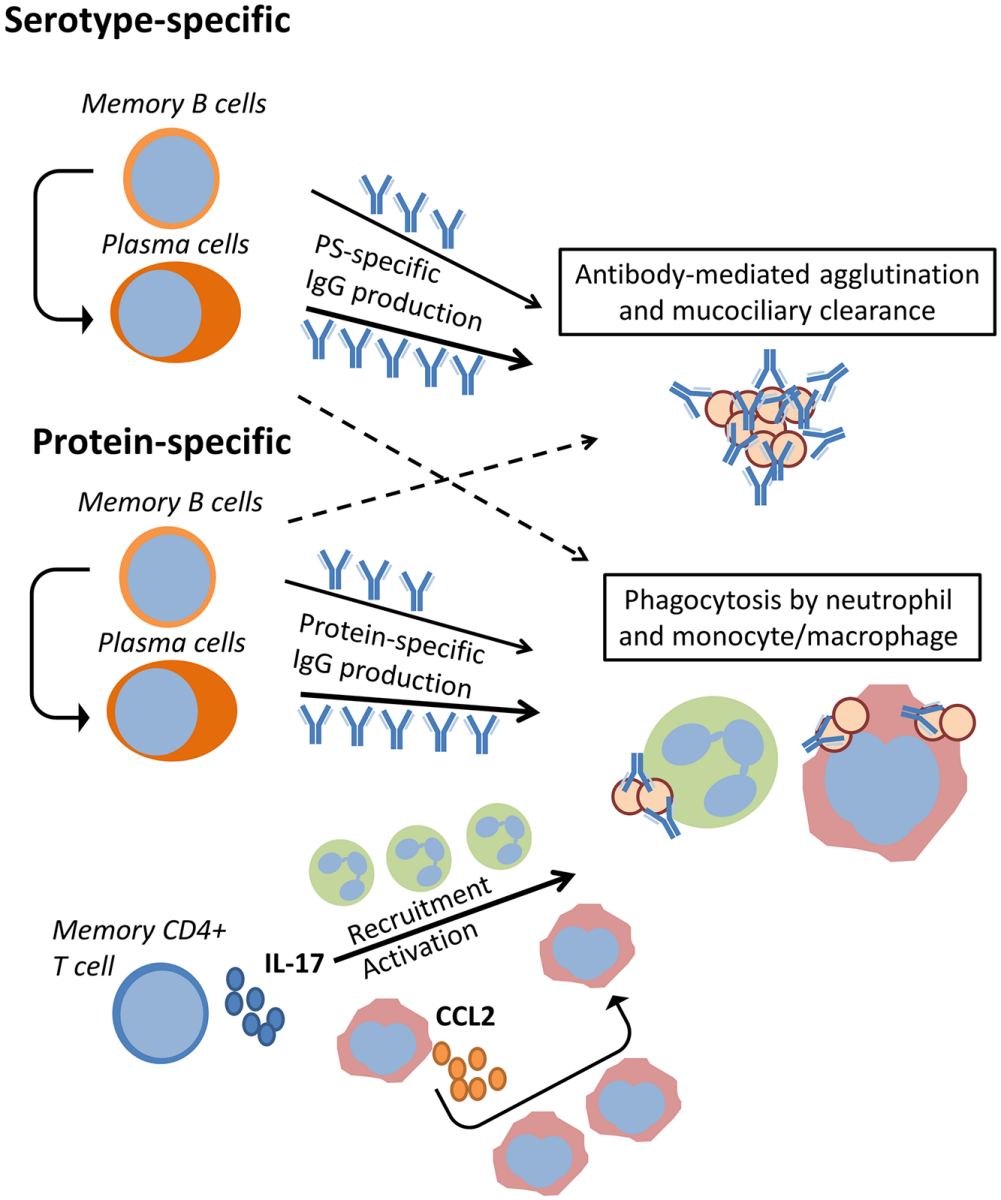
Immunological mechanisms of control of pneumococcal carriage. The serotype-dependent and -independent immunological mechanisms that control pneumococcal carriage are depicted. Memory B cells and plasma cells specific to capsule and proteins produce IgG. This can lead to antibody-mediated pneumococcal agglutination and antibody-mediated phagocytosis by neutrophils, monocytes, and macrophages. Moreover, IL-17A produced by memory CD4^+^ T cells might lead to recruitment and activation of neutrophils and monocytes/macrophages, thus increasing phagocytosis. CCL2, C-C motif chemokine ligand 2; CD4^+^, cluster of differentiation 4; IgG, immunoglobulin G; IL-17A, interleukin 17-A.

There is a large amount of evidence supporting the role of capsule-specific antibodies in preventing colonization. Pneumococcal colonization in adults confers virtually complete protection against acquisition of the same strain up to one year later in a controlled human infection model [19]. In contrast, healthy adults who were recently naturally colonized with a different serotype are not less susceptible to acquisition of experimental carriage [32]. This suggests that serotype-specific adaptive immune responses can control the establishment of carriage, although it cannot be excluded that strain-specific, serotype-independent memory responses protect against homologous reacquisition. Similarly, serotype-specific protection against carriage is present in toddlers for at least several serotypes, demonstrating that this plays a role not just in adults [33]. While capsule can be expressed at lower levels during colonization, its expression is required for colonization [34]. Importantly, increased amounts of capsule are associated with transmission in an infant mouse model of transmission [35]. Serotype-specific protection from acquisition of carriage is likely conferred by antibody-mediated bacterial agglutination on the mucosal surface that may aid mucociliary clearance and, as a result, prevent stable establishment along the epithelium (Fig 1) [36]. Agglutination occurs though the bi- or multivalency of immunoglobulin and has been shown to be independent of the immunoglobulin fragment crystallizable (Fc) region and interactions with complement [37]. A secreted pneumococcal protease can specifically cleave human IgA1, the most abundant antibody subtype on the mucosal surface of the nasopharynx, preventing IgA1-dependent agglutination [37]. This protease, however, does not target IgG. Antibodies can also act by facilitating complement-mediated opsonophagocytosis by effector cells and thus prevent acquisition or mediate clearance (Fig 1) [38]. Therefore, capsule-specific immunity can effectively prevent establishment of colonization.

Vaccination with PCV leads to an increase in vaccine-serotype–specific IgG, which accesses the mucosal surface [24]. Indeed, very high levels of capsule-specific IgG induced by PCV13 vaccination protect healthy adult volunteers from experimental carriage acquisition [39]. As expected following efficient blocking of carriage acquisition and transmission, the serotypes covered in PCV have been rapidly depleted in highly vaccinated communities in Europe and the United States [27, 40]. An exception is serotype 3, which has persisted longer than other serotypes following PCV introduction, perhaps because of its capacity to release its capsule and thus circumvent the effect of anticapsular IgG [41, 42].

For antibodies to confer direct protection against acquisition, higher anticapsular antibody levels (as induced by PCV) may be required than to protect against invasive disease [43]. Antibody titers greater than 0.35 ug/mL following PCV vaccination are associated with effective protection against invasive disease in infants [44, 45]. However, to confer protection against colonization, antibody titers greater than 4.0 ug/mL might be required, depending on serotype [43, 46]. A recent study assessing anticapsular levels in PCV-vaccinated toddlers in Kenya was unable, however, to identify protective cutoff levels against carriage [47]. This could explain the limited correlation found between serum levels of capsule-specific antibodies and acquisition in children and adults [9, 33]. Indeed, levels of polysaccharide-specific memory B cells but not IgG correlate with protection from experimental carriage acquisition [19, 32]. Such memory B cells can quickly differentiate into plasma cells, which can produce antibodies, following pneumococcal encounter and thus prevent acquisition (Fig 1) [32]. In addition, while capsule-specific responses are undoubtedly involved in controlling colonization, responses to other pneumococcal antigens, including surface proteins, likely participate in conferring protective immunity against carriage.

## Capsule-independent immunity

Longitudinal follow-up of children from birth to one year has demonstrated that carriage protects against subsequent carriage events also in a serotype-independent manner [48]. Indeed, this protection is observed prior to the maturation of capsule-specific antibodies, which are not observed until two years of age. Moreover, the duration and density of carriage episodes decrease with age and previous pneumococcal exposures [49, 50]. Epidemiological data and mathematical modeling have attributed such decreases predominantly to the gradual accumulation of serotype-independent immunity [51]. Models taking both serotype-independent and serotype-dependent immunity into account were able to accurately predict serotype frequency, diversity, and carriage duration. In contrast, models that only included serotype-specific immunity predicted a lower serotype diversity, as well as lower carriage rates in adults, compared with reported data. Several immunological mechanisms could explain such a serotype-independent mode of control, including antibodies directed against proteins, T helper type 17 (Th17) memory cells, and trained innate immune responses.

## Protein-specific antibodies

The role of antibodies against pneumococcal proteins in protection from carriage acquisition has been studied repeatedly with conflicting results, particularly in regards to human testing. In a study using a 23F-type human challenge model, increased baseline antibody levels against pneumococcal surface protein A (PspA) were found in individuals protected against acquisition of carriage [18]. Moreover, a recent study in Native American communities found that decreased antibody titers against PspC group 3 were associated with increased colonization by pneumococci expressing this variant [52]. However, because this study did not assess the relationship between antibody levels and carriage in a prospective manner, no conclusions on the protective potential of variant-specific antibodies could be drawn. In contrast, in studies using a 6B-type human challenge model, serum IgG levels against 18 pneumococcal proteins, including PspA and PspC, did not correlate with protection against carriage [19, 32]. However, the immunodominant N-terminus of PspA is highly variable, and it is possible that variant-specific immunity could be necessary to confer protection. A similar lack of correlation between protein-specific antibodies and carriage acquisition was observed in an observational study in young children [53]. Antibodies and mature B cells were found to be dispensable for the clearance of carriage in adult mice [54]. Recently, a vaccine inducing protein-specific antibodies was tested for its effect on carriage in a phase-2 clinical trial in infants in The Gambia [55]. This vaccine contained PCV10 combined with pneumolysin and pneumococcal histidine triad protein D (PhtD), and antibodies against both proteins were potently induced. However, this vaccine only conferred a 0.5% to 2.1% protection against non–vaccine-type (NVT) carriage, depending on dosage [55]. Because this study was designed to look at NVT acquisition, there was no cohort that did not receive any pneumococcal vaccine. Consequently, the effect of vaccination on VT acquisition could not be compared to natural colonization. In all groups, VT acquisition was around 10% in each 3- to 4-month interval in the year post vaccination (2–5 months old, 5–9 months old, 9–12 months old).

While difficult to assess, it is conceivable that high levels of antibody to a single antigen might be insufficient, while a cumulative effect of antibodies to several antigens could confer protection against carriage.

## Th17 responses

Murine models have indicated a role for CD4^+^ Th17 cells, which produce interleukin-17A (IL-17A), in controlling carriage density and duration following either vaccination with pneumococcal whole-cell vaccine (WCV) or repeated homologous colonization [56–58]. However, these findings have not yet been conclusively corroborated in humans.

A polymorphism in the *IL17A* gene was found to be associated with lower serum levels of IL-17 and increased carriage levels in young children [59]. Levels of pneumococcus-specific CD4^+^ Th17 cells and regulatory T cells (Treg) have been extensively studied in lymphoid tissues in children [60–64]. The ratio of Th17/Treg is low in children and increases with age as colonization frequency decreases [62]. In addition, this ratio is lower in colonized children than in uncolonized children [56, 57]. However, the association between the ratio of Th17/Treg and carriage state could also reflect the fact that carriage itself modulates the frequency of Th17 and Treg cells. Indeed, carriage increases Treg frequency in mice, which is associated with stable colonization [65]. Moreover, the finding that children in Bangladesh, where pneumococcal carriage and infection rates are relatively high, have increased Th17 responses compared with children in Sweden (where the incidence of carriage and infection with pneumococcus is much lower) would suggest that these responses alone are not sufficient in conferring protection [66].

Recently, the GEN-004 vaccine, consisting of three antigens identified following a proteomic screen for proteins that elicit robust Th17 cell responses (*Streptococcus pneumoniae* proteins SP0148, SP1912, and SP2108), was tested using a human challenge model [67]. This vaccine consistently reduced carriage acquisition by 18% to 36% versus placebo, but the differences were not statistically significant because the study was powered to detect 50% protection [67]. There was no evidence of reduced colonization density or duration up to day 14 following inoculation [67]. One limitation of the GEN-004 study is that Th17 cellular responses have not yet been reported, which limits the conclusions one can draw from the study.

## Neutrophils

In mice, the control of established colonization and clearance by Th17 cells depends on both neutrophils and macrophages (Fig 1) [56, 57]. Immunization with WCV leads to neutrophil recruitment to the nasopharynx following colonization, and depletion of these cells partially abrogates the protective effect of vaccination [56]. Such neutrophil recruitment is necessary to control carriage because neutrophils are not detected in the nasal mucosa of naïve mice [57]. Therefore, it is not surprising that the depletion of neutrophils did not increase pneumococcal acquisition in an infant mouse model of pneumococcal transmission [68]. In contrast to the case in mice, neutrophils are present in the nasal lumen of human adults and children [69, 70]. Therefore, neutrophils might be able to prevent the establishment of colonization in humans, while the effect of additional neutrophil recruitment is unclear.

In addition to mediating neutrophil recruitment, IL-17A increases the capacity of human neutrophils to kill pneumococci in vitro (Fig 1) [56, 69, 70]. Indeed, the vaccination of mice using a type 2 pneumococcal *pep27* mutant strain led to increased capacity of neutrophils to kill pneumococci in vitro [71]. This vaccination provided protection against colonization by a range of heterologous serotypes. The role of neutrophils against carriage in humans is supported by the fact that the capacity of a given serotype to resist neutrophil- (and possibly complement-) mediated killing in vitro is associated with its prevalence [72]. However, in a recent study of mouse cocolonization with six pneumococcal serotypes, the depletion of neutrophils did not alter relative serotype recovery [73]. It should be noted that this experiment was conducted using naïve mice that have delayed neutrophil recruitment dynamics [57, 73].

## Monocytes/Macrophages

Recolonization of mice by the same pneumococcal strain also increases levels of macrophages in the nasopharynx, albeit with delayed recruitment kinetics compared with neutrophils [57]. In mice, this recruitment of mucosal macrophages correlates with dynamics of clearance—a process that requires weeks to months. Macrophage depletion increases carriage density and delays carriage clearance in this model [57]. Molecular studies of this macrophage recruitment have identified innate and adaptive receptors and cytokines in a two-phase recruitment (Fig 1). Toll-like receptor 2 (TLR2) and IL-17A but not neutrophils or CD8^+^ T cells are required for this macrophage recruitment [57]. Indeed, TLR2 but not TLR4 was previously found to be required for pneumococcal clearance in mice [74]. Early recruited macrophages sense lysozyme-digested pneumococcal peptidoglycan through nucleotide-binding oligomerization domain-containing protein 2 (Nod2), produce C-C motif chemokine ligand 2 (CCL2), and induce recruitment of additional macrophages in a positive feedback loop [75]. Deletion of both TLR2 and Nod2 has a more profound effect on pneumococcal clearance than either alone, suggesting that these mechanisms are complementary [75].

Recently, the delayed clearance dynamics in infant mice compared with adult mice were linked to a failure to recruit monocytes to the nasopharynx, related to microbiota-induced tonic CCL2 production [76]. Similarly, elderly mice have dysfunctional macrophages and display impaired monocyte recruitment to the nasopharynx, which is associated with decreased clearance [77, 78]. In addition, prior influenza infection impairs monocyte/macrophage recruitment through type 1 interferon signaling, leading to increased colonization density in mice [79].

Unlike neutrophils, monocytes/macrophages are virtually absent from the human nasopharynx in uncolonized individuals [70]. Human macrophages express IL-17R, which can directly mediate recruitment following IL-17A encounter [80]. Whether these cells are recruited following pneumococcal carriage in adults or children has not been studied. However, the recruitment of monocytes to the nose during influenza infection is seen in young children, along with increased CCL2 levels in nasal aspirates [81].

IL-17A can also activate macrophages because the capacity of human alveolar macrophages to kill pneumococcus in vitro is increased by coculture with IL-17A (Fig 1) [20]. Interestingly, Th17-mediated immunity is also elicited by vaccination with whole-cell *Bordetella pertussis* but not by the acellular pertussis vaccine, boosting macrophage function [82]. Indeed, induction of *IL17* expression in baboons vaccinated with whole-cell pertussis but not acellular pertussis associates with protection against colonization [83]. In mice, wild-type influenza predisposes to pneumococcal pneumonia in part by impairing the phagocytic capacity of monocytes/macrophages by interferon gamma (IFNɣ)-mediated modulation of the scavenger receptor Macrophage Receptor With Collagenous Structure (MARCO) [84]. While the role of monocytes/macrophages in controlling human colonization has not been addressed directly, it is interesting that live-attenuated influenza vaccination (LAIV) increases carriage density in children [85].

## Effect of inflammation and other innate mechanisms

The contribution of other immunological modalities to the control of pneumococcal colonization has not been extensively studied and remains unclear. The presence of local inflammation is likely to predispose to carriage acquisition. Induction of inflammation by treatment with bacterial lipopolysaccharides (LPS) increases carriage acquisition in infant mice [68]. Furthermore, inflammation of the mucosa is associated with increased carriage levels in children [86]. Similarly, asymptomatic upper respiratory tract virus infection predisposes to experimental carriage acquisition in humans [87]. It is not clear to what extent respiratory viral infection and inflammation promote increased carriage by impairing innate immune responses, including neutrophil and monocyte function [84, 88]. Coinfection with virus may act through other mechanisms, such as (i) impairment of mucociliary clearance, (ii) up-regulation of pneumococcal receptors such as platelet-activating factor receptor (PAFr) and polymeric immunoglobulin receptor (pIgR) on epithelial cells, (iii) epithelial denudation and adherence to the exposed basal layer, and (iv) increased availability of nutrients such as sialic acid [89–93].

Finally, genetic ablation of the IFNa receptor increases colonization density in a murine model while not impairing recruitment of neutrophils and monocytes [94]. This provides further evidence that type 1 interferons might be involved in controlling carriage through induction by bacteria. The role of other immune cells, such as innate lymphoid cells, in controlling pneumococcal colonization has not been extensively studied [95].

## Future perspectives

A better understanding of the immunological factors that govern pneumococcal acquisition, control density, and mediate clearance will guide the informed development of novel antipneumococcal interventions.

The antibody-independent factors that control carriage acquisition following pneumococcal encounter still remain unclear. The recent development of murine transmission models and use of experimental human challenge models may allow new perspectives and further elucidation of these mechanisms [68, 96].

While a clear picture has emerged on how established pneumococcal colonization is controlled in mice, much of these data have not yet been validated in humans. Further studies of levels of neutrophils and monocytes following pneumococcal carriage should be performed to fill these gaps. In particular, the lack of additional recruitment of these cells in children may explain their high carriage levels. Moreover, longitudinal data on Th17/Treg levels and carriage presence and duration are required to determine causality in the interaction between these cells and carriage. Recently, anti–IL-17A monoclonal antibodies were licensed for treatment of psoriasis [97]. The carriage levels in individuals receiving such antibodies compared with controls would be an excellent tool to study the effect of IL-17A on colonization in a human setting. Pneumococcal genes that can be recognized by Th17 cells are under lesser diversifying selection than genes that are recognized by antibodies, which could make them excellent targets for vaccination [98].

Finally, a better understanding of the pneumococcal proteins that mediate serotype-independent immunity in humans should be identified. Several studies of pneumococcal specific protein antibody responses in children and adults have been unable to demonstrate an association with carriage [19, 32, 53]. And while recent protein-specific vaccine trials have shown only modest effects on colonization in randomized clinical trials (RCTs), the ecological effects of such protection are hard to predict [55, 67, 99]. In addition, these studies have examined only a few candidate antigens; ongoing trials of the WCV, which will examine impact on carriage as well as invasive disease, may provide further information in this regard. A wider array of antibody responses, as may be generated in response to WCV, could be required for non–serotype-specific protection.

## Conclusions

The immunological mechanisms that mediate serotype-dependent control of carriage have been well described, with capsule-specific memory B cells and IgG being able to prevent colonization through antibody-mediated agglutination and disease through opsonophagocytosis (Fig 1). However, the serotype-independent immune responses that are able to control pneumococcal carriage remain uncertain, especially in humans. It is currently hypothesized that Th17-mediated protein-specific responses play a role in controlling established carriage density and duration through recruitment and activation of neutrophils and macrophages. However, these data are largely derived from studies in adult mice with little human validation to date. Moreover, the array of pneumococcal proteins that would optimally confer such protection are still unidentified. There is also conflicting evidence from human studies for the capacity of protein-specific antibodies to confer protection. Therefore, further studies assessing these mechanisms in humans are necessary to foster the development of more broadly acting, serotype-independent vaccines.
